# A Comprehensive Equilibrium Analysis of Tartronate with Proton and Major Cations in Natural Fluids

**DOI:** 10.3390/molecules30071497

**Published:** 2025-03-27

**Authors:** Gabriele Lando, Clemente Bretti, Paola Cardiano, Anna Irto, Demetrio Milea, Concetta De Stefano

**Affiliations:** Department of Chemical, Biological, Pharmaceutical and Environmental Sciences, University of Messina, Viale Ferdinando Stagno d’Alcontres, 31, I-98166 Messina, Italy; glando@unime.it (G.L.); pcardiano@unime.it (P.C.); airto@unime.it (A.I.); dmilea@unime.it (D.M.); cdestefano@unime.it (C.D.S.)

**Keywords:** tartronic acid, protonation constants, ionic strength, thermodynamic modeling, Specific Ion Interaction Theory (SIT), weak complex formation, metal cations

## Abstract

This study presents a detailed thermodynamic investigation on the protonation behavior of tartronic acid in aqueous solutions of various ionic media, including sodium chloride, potassium chloride, tetramethylammonium chloride, and tetraethylammonium iodide. Specifically, potentiometric measurements were performed at temperatures ranging from 288.15 to 310.15 K and ionic strengths between 0.1 and 1.0 mol dm^−3^ to determine stoichiometric protonation constants in different ionic media. The formation of weak complexes between tartronate and alkaline metal cations was obtained by means of the Δ*pK* method. Moreover, data were modeled using the Debye–Hückel equation and Specific Ion Interaction Theory (SIT), allowing for the calculation of standard thermodynamic parameters and the assessment of the dependence of protonation constants on ionic strength. Additionally, the protonation behavior of tartronic acid was compared with that of structurally related acids, such as malonic and mesoxalic acids, providing insights into the role of molecular structure in acid dissociation. The results emphasize the significant role of entropic contributions in the protonation process and provide a comprehensive model for the thermodynamic properties of tartronic acid across a wide range of experimental conditions.

## 1. Introduction

**Scheme 1:**
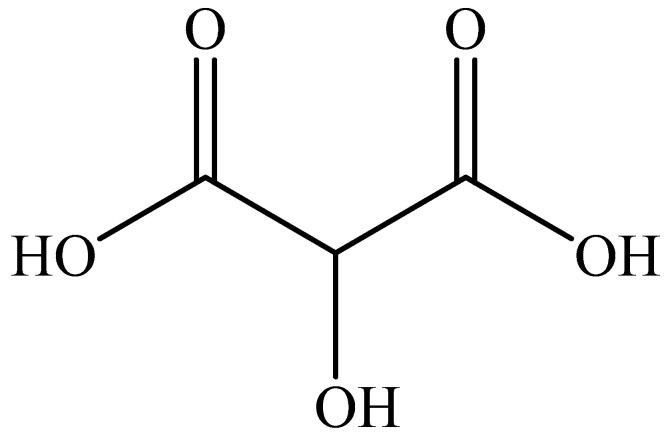
Structure of tartronic acid (H_2_L).

Tartronic acid (TA, L^2−^, Scheme 1) plays a vital role in plant and animal metabolism, functioning both as an intermediate and a regulator in various metabolic pathways [1,2,3,4]. Furthermore, it naturally occurs in various plant species [5,6,7]. It is known to inhibit the transformation of carbohydrates into fat under fat-deficient conditions, though it can also promote lipogenesis, as reported by Shi et al. [8]. As a key derivative of glycerol, tartronic acid is highly valued in the fine chemical industry [9,10,11]. It is extensively used in the synthesis of fine chemicals and novel polymers, offering a sustainable alternative to fossil-derived olefins and carboxylic acids in polymer production [12,13,14].

Beyond its biochemical roles, TA is employed as an anti-corrosive and protective agent, preventing oxidative degradation in the food industry and reducing corrosion in boilers and other high-temperature environments [15]. Its increasing significance in medical and industrial applications is exemplified by recent studies that have highlighted its potential as a highly efficient inhibitor of calcium oxalate crystallization [16]. In particular, the authors showed that the complexation between tartronic acid and Ca^2+^ at experimental concentrations is minimal, whereas citric acid sequesters about half of the free Ca^2+^ at the same concentration. This suggests that TA primarily inhibits calcium oxalate monohydrate crystallization through interactions with crystal surfaces rather than forming complexes with Ca^2+^ in a solution.

Research on tartronic acid capability to form complexes with metal cations, both regarding the synthesis and isolation of these complexes [17] as well as their behavior in aqueous solution [18], is not extensively covered. The tartronate anion has five potentially coordinating oxygen atoms [19], making it a promising chelator for metal cations.

From a thermodynamic perspective, particularly regarding the determination of stability constants of tartronic acid with various metal cations in aqueous solution, few studies are available. These are typically conducted at a single temperature and ionic strength. Campi et al. [18] reported data for a series of divalent metal cations (Ba^2+^, Ca^2+^, Mg^2+^, Ni^2+^, Co^2+^, Zn^2+^, Cu^2+^, and Cd^2+^) at *I* = 0.1 mol dm^−3^ (in NaClO_4_) at *T* = 293 K. Micskei et al. [20] determined pH metrically, at *I* = 1 mol dm^−3^ KCl at *T* = 298.15 K, the complexes of tartronate with Cr^2+^. Kirishima et al. [21] used potentiometric and calorimetric titration techniques to determine the thermodynamic quantities (Δ*G*, Δ*H*, and TΔ*S*) of U(VI) complexation with tartronate. Mukerjee et al. [22] reported data for Mn^2+^, Ni^2+^, and Cu^2+^, whereas Manning et al. [23] focused on Eu^3+^. However, these values did not meet the selection criteria according to the NIST database [24].

Regarding protonation constants, the only available data are from Campi et al. [18] at *I* = 0.1 mol dm^−3^ (in NaClO_4_) at *T* = 293 K. Stepwise protonation enthalpy changes (∆*H*^0^) were determined via calorimetric titration by Kirishima et al. [21] at *I* = 1 mol dm^−3^ in NaClO_4_ at *T* = 298.15 K.

The aim of this paper is to determine the protonation constants of tartronate in aqueous solutions of NaCl, KCl, (CH_3_)_4_NCl, and (C_2_H_5_)_4_NI by means of potentiometric measurements under different experimental conditions, including the reagent concentration, ionic strength, and temperature. Whenever possible, instrumentation was varied by using different glass and reference electrodes, as well as reagents from different batches, to minimize potential systematic errors, such as those caused by electrode calibration issues, thereby increasing the reproducibility of the experiments.

In this context, it is particularly important to emphasize that the determination of reliable thermodynamic parameters and the subsequent development of robust models depend heavily on the quality of the experiments, which must eliminate all potential sources of systematic error and ensure reproducibility. Once determined, protonation constants were modeled as a function of ionic strength and temperature to obtain data under standard conditions (e.g., infinite dilution) and formation constants of weak ion pairs between tartronate and alkaline metal cations of the supporting electrolyte using the Δ*pK* method and ionic strength dependence parameters (e.g., specific ion interaction coefficients). The above-mentioned parameters enable the calculation of equilibrium constants at any temperature and ionic strength within the range considered using established equations, such as the Debye–Hückel equation and the Specific Ion Interaction Theory (SIT). All the determined thermodynamic data could be useful for potential future investigations aimed at testing tartronic acid capability in environmental chemistry or pharmacology.

## 2. Results and Discussion

**Figure 1:**
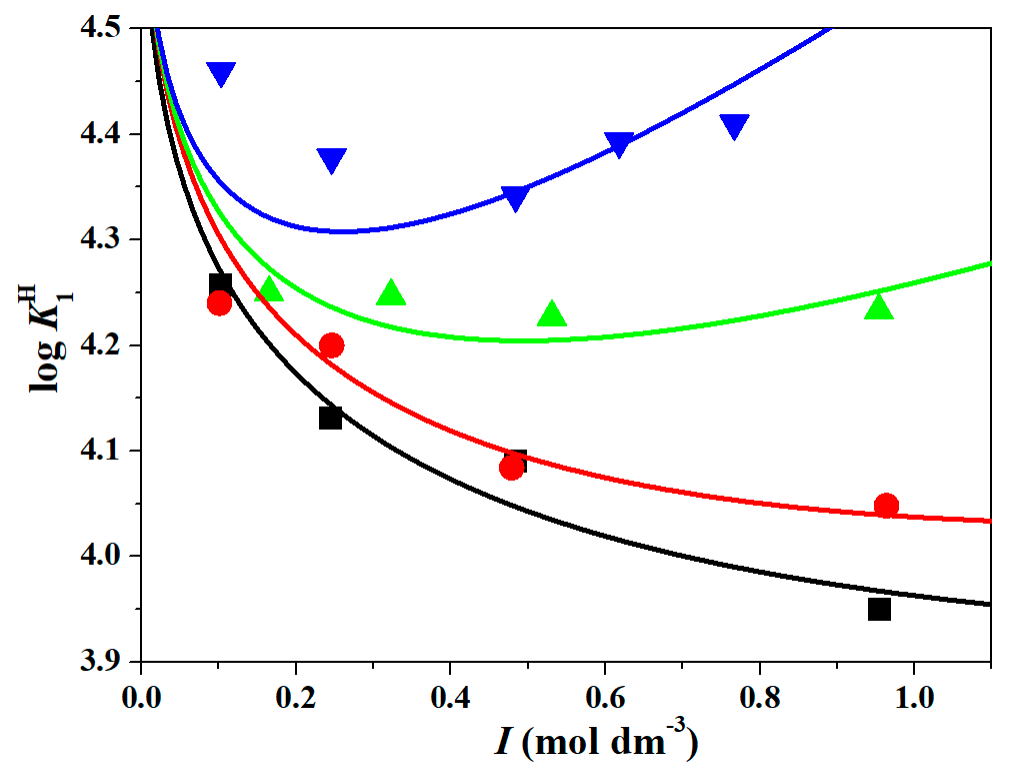
Dependence of the first protonation constants log $K_{101}^{H}$ on ionic strength in different ionic media at *T* = 288.15 K: ∆ (CH_3_)_4_NCl, ▽ (C_2_H_5_)_4_NI, ○ KCl, and □ NaCl.

The protonation constants of tartronate were determined potentiometrically in NaCl_(aq)_, KCl_(aq)_, (CH_3_)_4_NCl_(aq)_, and (C_2_H_5_)_4_NI_(aq)_ at various temperatures and ionic strengths. The results obtained for the first and second protonation constants are provided in Table 1 in the molal concentration scale (complete experimental data, together with associated errors, are available in Tables S1–S4). As an example, the trend of the first tartronate protonation constant as a function of ionic strength is illustrated in Figure 1 at *T* = 288.15 K, together with the model equation according to the SIT approach (solid colored lines). A similar pattern is observed at other temperatures, although with greater deviations. The goodness of fit criteria suggested by Meloun [25] and Gans [26] were used to evaluate results obtained. As an example, for data obtained in NaCl at *T* = 298.15 K (a total of eleven titrations), the arithmetic mean of residuals (the residual bias, the difference between calculated and measured *e.m.f.* values) resulted in 0.087 mV, which is not statistically different from the theoretical value of 0 mV. The mean of the absolute value of residuals resulted in 0.3 mV, which is reasonably close to the uncertainty in the measured *e.m.f.* value, namely, 0.15 mV; skewness was 0.9, and kurtosis was 3.5, which indicate that residuals are reasonably symmetric and with a slightly positive kurtosis.

The protonation constants of the ligand TA (L^2−^ in its fully deprotonated form) and the formation constants of weak complexes refer to the following equilibrium:

*i* H^+^ + *j* X^+^ + *k* L^2−^ = H*_i_*X*_j_*L*_k_*^(*i* + *j* − 2*k*)^         *K*_*ijk*_ (1)

where X^+^ can be Na^+^ or K^+^. If j = 0, equilibrium (1) refers to a protonation constant (instead of *K*_i0k_, they are reported as $K_{i0k}^{H}$).

As observed, the protonation constant values decrease as both the ionic strength and temperature increase. Additionally, the data in NaCl are noticeably lower than those in KCl, (CH_3_)_4_NCl, and (C_2_H_5_)_4_NI. This difference can be attributed to different activity coefficients of proton and tartronate species in different ionic chemical environments or to the formation of weak ion pairs involving the cations of the supporting electrolytes (X⁺) and the anionic tartronate species (L^2−^ and HL^−^ species). When comparing the protonation constants obtained with different electrolytes, the Na⁺ cation, being the smallest one, interacts more readily with the ligand. Consequently, the protonation equilibrium shifts toward the reactants, resulting in a lower conditional constant value. As the cation size increases (i.e., K⁺ → (CH_3_)_4_N⁺ → (C_2_H_5_)_4_N⁺), steric hindrance becomes more significant, reducing the likelihood of cation interactions with tartronate. This, in turn, causes the protonation equilibrium to shift less toward the reactants, leading to higher conditional protonation constants.

While only two protonation constants are reported in the tables, the formation of a third species was observed under specific conditions (i.e., in NaCl at *T* = 288.15 and 298.15 K) corresponding to the following equilibrium:

L^2−^ + HL^−^ = HL_2_^3−^ (2)

This species could be tentatively described, assuming a proton acting as a bridge between two fully deprotonated tartronate molecules through the hydroxylate groups. However, the existence of this species is not documented in the literature, making a conclusive assessment difficult. Furthermore, the formation of this species was not observed under all experimental conditions. For this reason, the formation of the HL_2_^3−^ species cannot be definitively proved unless dedicated measurements are performed. Nevertheless, some of the results obtained in NaCl_(aq)_ are provided in Table 2.

The tabulated values indicate that the stability of the HL_2_^3−^ species is relatively low, slightly exceeding a decimal logarithm value of 1.0. Additionally, the stability of this species appears to increase with ionic strength, suggesting that the Na⁺ cation may contribute to shielding the negative charges of the two tartronate molecules. This hypothesis helps to explain the difficulty in refining the stability of the HL_2_^3−^ species in ionic media containing bulkier cations (e.g., tetraalkylammonium).

When HL_2_^3−^ is excluded, the results from fitting the experimental data in the molal concentration scale to Equation (3) (see Section Materials and Methods) are summarized in Table 3 (SIT model). Corresponding data in the molar concentration scale (the EDH model; more details are given in the Supplementary Materials) are given in Table S5.

Furthermore, by expressing $\Delta \varepsilon_{i01}$ in terms of the species involved in the equilibria, as shown in Equation (S4), the individual specific interaction coefficient involving the tartronate species can be obtained, provided that the interaction coefficient of the proton with anions of the supporting electrolyte and the activity coefficient of the neutral H_2_L^0^_(aq)_ species, computed by multiplying the Setschenow constants (*k_m_*) by ionic strength, are known. In this case, *ε*(H^+^, Cl^−^) and *ε*(H^+^, I^−^) were taken from the literature [28], whereas the Setschenow coefficient is unknown. Typically, the *k*_m_ value is experimentally determined through solubility measurements or solvent partitioning under varying electrolyte concentrations (e.g., NaCl). Some quick tests for both measurement types evidenced that the water solubility of tartronic acid is too high. During solubility experiments, the possible formation of dimeric or higher species cannot be excluded, while octanol/water partitioning measurements yielded negative results, as all the tartronic acid dissolved in water.

For these reasons, the Setschenow coefficient of the neutral H_2_L^0^_(aq)_ species was predicted by the regression tree chemometric algorithm proposed by De Stefano et al. [29], and it was considered to be valid in all ionic media. In this way, the unknown specific interaction coefficients can be obtained by fitting experimental data, in the molal concentration scale, to Equations (S3) and (S4) using LIANA software, and the results are reported in Table 4.

The dependence of protonation constants on ionic strength in different ionic media has also been interpreted in terms of the formation of weak complexes between the cations of the supporting electrolyte and the anionic ligand species [30,31,32,33]. In this study, data were analyzed using ES2WC software, which enabled the determination of the formation constants of weak complexes, as well as the effective protonation constants (Table 5).

The constant values of the weak ion pairs reveal that they decrease as the size of the cation increases (log ^T^*K*^Na+^ > log ^T^*K*^K+^ > log ^T^*K*^(CH3)4N+^), a trend that is consistent with the previously observed variation in protonation constants across different ionic media.

A comparison of the protonation constants of tartronate with those of malonate (mal) and mesoxalate (mesox), as presented in Table 6, reveals that the p*K*a values for malonate are higher than those for tartronate, while mesoxalate exhibits lower p*K*a values. This results in the following trend: malonate > tartronate > mesoxalate.

Differences can be attributed to the molecular structure of the three ligands, which features a different oxidation state of the central carbon atom. Compared to tartronic acid, malonic acid lacks the hydroxyl group bound to the central carbon atom, resulting in less acidic carboxylic hydrogen atoms. Conversely, mesoxalic acid displays a carbonyl group bond to the central carbon atom, which exerts an electron-withdrawing effect. As a result, the hydrogen atoms of the carboxylic groups of mesoxalate are more acidic than those of tartronic acid. Analyzing the data presented in Table 6, it is clear that the first protonation constants differ by approximately one logarithmic unit, favoring malonate across all ionic strengths. For the second protonation constant, the difference is approximately 0.5–0.6 logarithmic units, again favoring malonate.

Regarding comparisons of tartronate protonation constants, no significant differences are observed between the values reported in the literature (Table 6, log$\;K_{101}^{H}$ = 4.24 and log$\;K_{201}^{H}$ = 2.02 at *I* = 0.1 and *T* = 293 K in Na^+^ medium) and those experimentally determined in this study (log $K_{101}^{H}\;$= 4.29 and log $K_{201}^{H}$ = 2.08 under the same conditions).

Further comparisons between protonation constants and enthalpy change values of malonate and tartronate are presented in Table 7. Regarding enthalpy changes, both ligands exhibit similar values for the first protonation step. However, more significant differences are observed in the second step. Specifically, the enthalpy changes for tartronate are positive, indicating an endothermic process, whereas for malonate, they are only slightly negative.

**Figure 2:**
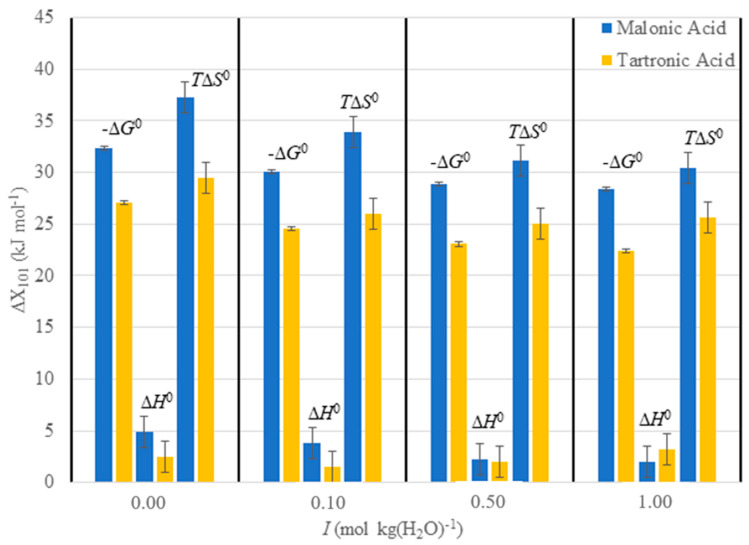
Thermodynamic parameters (${-\Delta G}_{101}^{0}$, ${\Delta H}_{101}^{0}$, and *T*${\Delta S}_{101}^{0}$, in kJ mol^−1^) for malonic acid and tartronic acid at *T* = 298.15 K. Error bars for tartronic acid are experimentally determined, whereas those of malonic acid are estimated.

In Figure 2, the thermodynamic parameters for the first step of protonation of malonate and tartronate are presented. As observed (the same findings apply to the second protonation step), the most significant contribution to stability is of an entropic nature. This is expected for the reaction between a proton and a carboxylate anion, primarily due to contributions such as charge neutralization and the displacement of water molecules (these factors also apply to the second protonation step).

## 3. Materials and Methods

### 3.1. Chemicals

All chemicals were purchased from Merck (Darmstadt, Germany) at the highest purity available and were used without further purification, except for tetramethylammonium chloride (CH_3_)_4_NCl and tetraethylammonium iodide (C_2_H_5_)_4_NI, which were re-crystallized from methanol as described by Perrin et al. [35]. Potassium hydroxide, sodium hydroxide, tetramethylammonium hydroxide, tetraethylammonium hydroxide, and hydrochloric acid solutions were prepared from concentrated solutions and standardized against potassium hydrogen phthalate (for bases) and sodium carbonate (for acids), previously dried in oven at *T* = 383.15 K for 2 h. The concentration of tartronic acid in the solutions was determined by alkalimetric titrations. Sodium and potassium chloride solutions were prepared by weighing the solids, previously dried in oven at *T* = 383.15 K for 2 h. Strong base solutions were stored in dark bottles, and soda lime traps were used to prevent the dissolution of carbon dioxide. All solutions were freshly prepared using grade A glassware and twice-distilled water (ρ ≥ 18 MΩ cm). A complete list of all chemicals used is provided in Table S6.

### 3.2. Apparatus and Procedure for the Potentiometric Measurements

To ensure the accuracy of the potentiometric measurements and minimize systematic errors due to instrumental issues, three potentiometric systems were employed: two Metrohm 809 Titrando units (Herisau, Switzerland) and one Thermo Fisher Orion Star T940 (Thermo Fisher Scientific, Waltham, MA, USA). The three systems were equipped with two different electrode setups: an Orion Sureflow combined glass electrode (model 8172, Thermo Fisher Scientific, Waltham, MA, USA) with an accuracy of ±0.15 mV and an Orion Ross-type non-combined glass electrode (model 8101, Thermo Fisher Scientific, Waltham, MA, USA) with a double junction reference electrode (Ag/AgCl in KCl, model 900200), with a system accuracy of ±0.15 mV.

The titrant was added using Metrohm 800 Dosino automatic burettes (Metrohm, Herisau, Switzerland), when 809 Titrando were employed, with an estimated accuracy of ±0.003 cm^3^, and the Orion (Thermo Fisher Scientific, Waltham, MA, USA) Star T940 10 cm^3^ burette, with an estimated accuracy of ±0.0004 cm^3^. Measurement vessels consist of double-walled cells that allowed the solution to be maintained at a constant temperature using a D1-G Haake thermostatically controlled cryostat (uncertainty ±0.1 K).

Both systems were interfaced with a PC using dedicated software (Metrohm TiAMO 2.5 and Orion T Star 900) to automatically acquire pairs of cm^3^ mV^−1^ values. Both software also allowed for setting titrant volume, density of recorded points, acceptable drift for point acquisition, and all other parameters related to the acid–base titration.

A magnetic stirrer in the first two systems and a mechanical stirrer in the second one ensured constant stirring speed to homogenize the solution. In the measuring cell, purified and pre-saturated N_2(g)_ was introduced to remove O_2(g)_ and CO_2(g)_ and prevent unwanted redox and acid–base reactions. Potentiometric measurements were conducted by titrating solutions containing tartronic acid, the supporting electrolyte, and hydrochloric acid, ensuring that predetermined values of ionic strength (ranging from 0.1 to 1.0 mol dm^−3^) and pH (generally ~ 2.0) with standard solutions of NaOH_(aq)_, KOH_(aq)_, (CH_3_)_4_NOH_(aq)_, and (C_2_H_5_)_4_NOH_(aq)_ until complete deprotonation of tartronic acid. The titrant was added in predetermined quantities based on the desired number of points to be recorded (generally about 70) and the optimal pH change between consecutive titration points. The experimental conditions adopted for the potentiometric measurements are reported in Table 8.

Calibration runs were performed by titrating solutions containing only the ionic medium and hydrochloric acid under the same ionic strengths and temperature conditions of the measurements with tartronic acid, with the aim of determining standard electrode potential (*E*^0^) and the acidic junction coefficient (*j*_a_,* E*_jb_ = *j*_a_ [H^+^]). In this way, the pH scale is the free scale pH = −log [H^+^], where [H^+^] is the free proton concentration (not activity). The reliability of the calibration in the alkaline range was checked by alternatively calculating the ionic product of water ionic water product (*K*_w_), alkaline junction coefficients (*j*_b_,* E*_ja_ = *j*_b_ [H^+^]), and slope of the electrode(s). In the pH range of our measurements (2.0 ≤ pH ≤ 11.0), *j*_a_ and *j*_b_ were negligible. All of the refined calibration parameters, reported in Tables S7–S10, were used to convert measured *e.m.f.* (*E*) to pH values as pH = (*E*^0^ − *E*)/59.16.

### 3.3. Calculations

All equilibrium constants reported in this paper are expressed in decimal logarithmic form. The solutions were prepared according to the molar concentration scale. Conversion between molarity and molality was performed using appropriate density values, as described in [27].

The processing of experimental data obtained potentiometrically was carried out using several computer programs. The BSTAC program version 7.3 [36] employs the nonlinear least squares method, allowing for the refinement of analytical parameters derived from potentiometric measurements at various ionic strengths. It allows for the calculation of both conditional and thermodynamic protonation constants, as well as the formation constants of complex species that may form from components present in solution. Additionally, BSTAC refines the parameters related to the dependence of constants on ionic strength.

The non-linear least square program LIANA (Linear And Nonlinear Analysis, version 30 December 1999) [36] was used to fit different equations. ES2WC software (version 10 September 1997) [37] enables the determination of stability constants for weak complexes by comparing the protonation constants obtained in one or more interacting ionic media with those from a non-interacting ionic medium using the “Pure Water model” [30,31,32,33]. Distribution diagrams were generated using PyES software version 2.0.15.beta [32].

The dependence of protonation constants on ionic strength was studied according to two approaches. The first one, based on a hybrid chemico-physical model, considers the variation of activity coefficients using the extended Debye–Hückel type equation (EDH) and the Specific Ion Interaction Theory [28,38,39,40]. The second approach is a purely chemical model, which considers the differences in the values of protonation constants obtained in different ionic media with the formation of weak species between tartronate and the ions of the supporting electrolyte using the so-called “Pure water” model [30,31,32,33].

A dedicated section containing all the equations used to derive the fitting ones is given in the Supporting Information. Here, it is relevant to underline that for the SIT approach, the use of the temperature-independent molal concentration scale is mandatory, and the fitting equation is reported in Equation (3), whereas for the second one, since it is based on the comparison of concentrations obtained in different ionic media, the use of the molar concentration scale is required, and the fitting equation is reported in Equation (8):

(3) $$\log\;K_{i0k}^{H}={{\log\;}^{T}K}_{i0k}^{H\;\theta }\;\;-\;\frac{z_{i0k}^{*}\;\cdot \;A\;\cdot \;\sqrt{I}}{1+1.5\;\cdot \;\sqrt{I}}+{\Delta \varepsilon }_{i0k}\;\cdot \;I+\frac{{\Delta^{T}H}_{i0k}^{0}\;-\;\frac{z_{i0k}^{*}\;\cdot \;A^{\prime }\;\cdot \;\sqrt{I}}{1+1.5\;\cdot \;\sqrt{I}}+{\Delta \varepsilon }_{i0k}^{\prime }\;\cdot \;I}{R\cdot \ln\;\left(10\right)}\cdot \left(\frac{1}{\theta }-\frac{1}{T}\right)\cdot 52.23$$

(4) $$z_{i0k}^{*}=\Sigma \;{{\left(\mathrm{charge}\right)}^{2}}_{\mathrm{react}}\;-\;\Sigma \;{{\left(\mathrm{charge}\right)}^{2}}_{\mathrm{prod}}$$

(5) $$A=\left(0.51\;+\;\frac{0.856\;\cdot \;\left(T\;-\;\theta \right)+0.00385\;\cdot \;{\left(T\;-\;\theta \right)}^{2}}{1000}\right)$$

(6) $$A^{\prime }=RT^{2}\cdot \ln\;\left(10\right)\cdot \frac{\partial A}{\partial T}=1.5+0.024\;\cdot \;\left(T\;-\;\theta \right)$$

(7) $${\Delta \varepsilon }_{i0k}^{\prime }=RT^{2}\cdot \ln\;\left(10\right)\cdot \frac{\partial {\Delta \varepsilon }_{i0k}}{\partial T}$$

where $K_{i0k}^{H}$ and Ki0kH θT are the stoichiometric (or conditional) and thermodynamic protonation constants at the reference temperature *θ* = 298.15 K, respectively, $\Delta \varepsilon_{i0k}$ is the summation of the specific interaction coefficients covering all the interactions between the species involved in the equilibrium and the counter ions of the supporting electrolyte; ${\Delta^{T}H}_{i0k}^{0}$ is the protonation enthalpy change at infinite dilution; 52.23 is equal to 1/R·ln10, and ${\Delta \varepsilon }_{i0k}^{’}$ accounts for the ionic strength dependence of protonation enthalpy.

(8) $$\log\;K_{\mathrm{ijk}}=\log^{T}\;K_{\mathrm{ijk}}+{A1}_{\mathrm{ijk}}\;\cdot \;\Delta T\;-\;z_{\mathrm{ijk}}^{*}\;\cdot \left(\frac{\sqrt{I}}{2\;+\;3\cdot \sqrt{I}}+I^{1.5}\;\cdot \;d_{1}\right)+I\;\cdot \;\left(c_{0}\cdot p_{\mathrm{ijk}}^{*}+{(c}_{1}+{A2}_{\mathrm{ijk}}\;\cdot \;\Delta T)\cdot z_{\mathrm{ijk}}^{*}\right)$$

(9) $$p_{ijk}^{*}=\sum {stoichiometric\;coeff.}_{react}-\sum {stoichiometric\;coeff.}_{prod}$$

where *c*_0_, *c*_1_, and *d*_1_ are the ionic strength dependence parameters valid for both effective protonation constants and weak complex formation constants; ∆*T* = *T* − 298.15 K, ${A1}_{\mathrm{ijk}}$, and ${A2}_{\mathrm{ijk}}$ represent temperature gradients for log ^T^$K_{\mathrm{ijk}}$ and *c*_1_, respectively.

## 4. Conclusions

**Figure 3:**
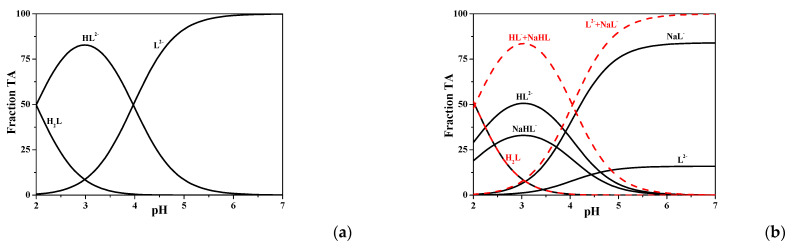
Distribution diagrams of the TA (c_L_ = 5 mmol dm^−3^) species as function of the pH in NaCl_(aq)_ at *I* = 0.7 mol dm^−3^ and *T* = 298.15 K according to the SIT (**a**) and the pure water (**b**) approaches.

In this study, potentiometric titrations were conducted under various conditions of ionic strength (0.1 < *I*/mol dm^−3^ ≤ 1.0), temperature (288.15 ≤ *T*/K ≤ 310.15), and ionic media (NaCl, KCl, (CH_3_)_4_NCl, and (C_2_H_5_)_4_NI) to study the influence of the variation of such chemico-physical variables on the apparent protonation constants of tartronate. The ionic strength dependence and the medium effect were interpreted in two different ways, namely, considering the SIT approach and the “pure water” model. Both interpret differences measured in the protonation constants obtained in different media, assuming a certain interaction between the species involved in equilibria and the ions of the supporting electrolyte. The SIT approach is a hybrid chemico-physical model that considers specific short-range interaction coefficients; the pure water one is a chemical model that postulates the formation of weak ion pairs. As a result, in the case of the SIT approach, the output is a list of specific interaction coefficients between ions of opposite charge (e.g., *ε*(L^2−^,Na^+^), *ε*(HL^−^,K^+^)) and protonation constants at infinite dilution, whereas in the case of the PW model, there are protonation constants at infinite dilution (that must be equal to those obtained to those of the SIT model), weak ion pair formation constants (e.g., log *K*_NaL^−^_ and log *K*_KHL_), and ionic strength dependence parameters equal to all species. However, even if the two models come from different assumptions, the modeling ability is often comparable in terms of chemical information carried. This can be demonstrated looking at Figure 3, where the distribution diagram of the H^+^/TA species is computed in a NaCl solution at *I* = 0.7 mol dm^−3^ (simulating ionic strength of seawater) and *T* = 298.15 K.

In diagram a, HL^−^ is the main species between pH ~ 2 and pH ~ 4, with a maximum of ~ 80% at pH ~ 3, and fully deprotonated tartronate is present at pH > 4. In diagram b, sodium species were also considered, and their formation percentage is quite high, as the NaHL^0^_(aq)_ is present between pH ~ 2 and pH ~ 5, reaching 30% at pH ~ 3, whereas NaL^−^ is the main species at pH > 4, achieving > 95% at pH > 6. The dotted red lines in diagram b represent the sum of the species containing tartronate at the same protonation degree. For example, HL^−^ is summed up to NaHL^0^_(aq)_, as well as NaL^−^ to L^2−^. Diagram b, drawn only with the dotted red curves, is equivalent to diagram a, as differences in the crossover points are about 0.03 pH units.

Formation constants of weak complexes with cations from different ionic media were also determined, identifying ML^−^ and MHL^0^_(aq)_ as the main species formed. The results suggest that the tartronate ligand interacts with cations in the order Na^+^ > K^+^ > (CH_3_)_4_N^+^ > (C_2_H_5_)_4_N^+^, which is attributed to differences in ion size.

Given the structural similarity of tartronic acid to mesoxalic and malonic acids, a comparison of their protonation constants was performed to explore potential correlations between numerical values and molecular structures, with a particular emphasis on the oxidation state of the central carbon.

Thermodynamic parameters, including entropy and enthalpy changes, were analyzed and compared with those of the malonate ligand, revealing that the spontaneity of the tartronate protonation process is predominantly driven by entropic contributions.

In conclusion, this work enhances the understanding of the thermodynamic properties of tartronic acid and defines its chemical speciation acid across a broad range of experimental conditions, including those relevant to natural and biological fluids.

## Figures and Tables

**Table 1:** Protonation constants of tartronate in different ionic media at different ionic strengths (on the molal concentration scale, mol kg^−1^ (H_2_O), and temperatures.

	NaCl			KCl			(CH_3_)_4_NCl			(C_2_H_5_)_4_NI		
*T*/K	*I*	$\log K_{101}^{H}$	$\log\beta_{201}^{H}$	*I*	$\log K_{101}^{H}$	$\log\beta_{201}^{H}$	*I*	$\log K_{101}^{H}$	$\log\beta_{201}^{H}$	*I*	$\log K_{101}^{H}$	$\log\beta_{201}^{H}$
288.15	0.102	4.26	6.363	0.101	4.24	6.227	0.168	4.24	6.264	0.105	4.45	6.56
288.15	0.246	4.13	6.176	0.248	4.20	6.094	0.335	4.23	6.289	0.258	4.35	6.49
288.15	0.488	4.09	6.072	0.486	4.08	5.908	0.564	4.20	6.308	0.532	4.30	6.47
288.15	0.972	3.94	5.875	0.991	4.04	6.076	1.064	4.19	6.266	0.697	4.34	6.41
288.15										0.893	4.34	6.45
298.15	0.113	4.27	6.346	0.095	4.29	6.425	0.172	4.23	5.913	0.104	4.38	6.55
298.15	0.249	4.12	6.224	0.248	4.15	6.220	0.336	4.23	6.207	0.256	4.38	6.59
298.15	0.470	4.05	6.060	0.492	4.02	6.046	0.574	4.14	5.825	0.258	4.33	6.45
298.15	0.934	3.93	5.823	0.990	3.96	5.774	0.792	4.22	6.266	0.532	4.39	6.60
298.15							1.071	4.18	6.513	0.641	4.38	6.55
298.15										0.892	4.38	6.49
310.15	0.103	4.26	6.360	0.103	4.30	6.472	0.169	4.28	6.349	0.105	4.43	6.70
310.15	0.248	4.12	6.100	0.249	4.15	6.231	0.333	4.19	6.183	0.255	4.40	6.64
310.15	0.491	3.97	5.856	0.493	4.05	6.051	0.510	4.21	6.258	0.534	4.30	6.44
310.15	0.982	3.96	6.128	0.993	4.09	6.163	1.075	4.19	6.219	0.896	4.41	6.53

The conversion of ionic strength and protonation constants was carried out by using the procedure reported in [27]. Standard uncertainties u: u(*T*) = 0.1 K; u(*K*) = 0.01; u(*p*) = 1 KPa.

**Table 2:** Protonation constants obtained in NaCl_(aq)_ at different ionic strengths, on the molal concentration scale, and temperatures considering the HL_2_^3−^ species.

*T/K*	*I * ^a^	$\log K_{101}^{H}$	$\log K_{201}^{H}$	$\log K_{102}^{H}$
288.15	0.00	4.68	2.30	0.58
288.15	0.10	4.25	2.08	0.99
288.15	0.25	4.11	2.00	1.11
288.15	0.50	4.00	1.93	1.17
288.15	1.00	3.90	1.85	1.19
298.15	0.00	4.70	2.24	1.07
298.15	0.10	4.26	2.03	1.42
298.15	0.25	4.12	1.96	1.43
298.15	0.50	4.00	1.92	1.33
298.15	1.00	3.89	1.88	1.02

^a^ mol kg^−1^ (H_2_O); relative expanded uncertainties u are expressed as 0.95 level of confidence. Standard uncertainties u are u(*T*) = 0.1 K; u(*K*) = 0.01; u(*p*) = 1 KPa.

**Table 3:** Parameters obtained fitting stepwise protonation constants to Equation (3) in the molal concentration scale (mol kg^−1^ (H_2_O)) at *T* = 298.15 K and *p* = 0.1 MPa.

			Molal Concentration Scale (SIT)				
			NaCl	KCl	(CH_3_)_4_NCl	(C_2_H_5_)_4_NI	
*i*	logKi01HT	ΔHi010T	$\Delta \varepsilon_{i01}$				$\Delta \varepsilon_{i01}^{\prime }$
1	4.74 ± 0.01 ^a^	2.3 ± 1.9 ^a^	−0.01 ± 0.02 ^a^	0.07 ± 0.03 ^a^	0.26 ± 0.02 ^a^	0.17 ± 0.02 ^a^	2.7 ± 3.0 ^a^
2	2.30 ± 0.01	2.7 ± 3.0	0.08 ± 0.04	0.08 ± 0.03	0.52 ± 0.02	0.32 ± 0.03	1.4 ± 1.5

^a^ 95% C.I.; standard uncertainties (*u*): *u*(*T*) = 0.1 K; *u*(*p*) = 1 kPa.

**Table 4:** Specific interaction coefficient *ε*_(*x*,*y*)_.

*x*	*y*	ε(x,y)	C.I. ^a^	Ref
H^+^	Cl^−^	(0.136 + (0.084 − 0.136)/(*I* + 1))	-	[28]
H^+^	I^−^	(0.173 + (0.204 − 0.173)/(*I* + 1))	-	[28]
Na^+^	L^2−^	−0.28	0.06	This work
K^+^	L^2−^	−0.20	0.06	This work
(CH_3_)_4_N^+^	L^2−^	0.09	0.05	This work
(C_2_H_5_)_4_N^+^	L^2−^	0.33	0.06	This work
Na^+^	HL^−^	−0.16	0.06	This work
K^+^	HL^−^	−0.16	0.05	This work
(CH_3_)_4_N^+^	HL^−^	−0.06	0.04	This work
(C_2_H_5_)_4_N^+^	HL^−^	0.01	0.05	This work
H_2_L^0^_(aq)_		−0.12 ^b^	-	[29]

^a^ 95%; ^b^ Setschenow coefficient *k_m_*.

**Table 5:** Ionic strength and temperature dependence parameters of weak complexes of tartronate with Na^+^ and K^+^ e (CH_3_)_4_N^+^ cations (Equation (8)).

Equilibrium	p*	z*	*i*	*j*	*k*	log ^T^*K_ijk_*	C.I. ^a^	A1*_ijk_*	C.I. ^a^
H^+^ + L^2−^ = HL^−^	1	4	1	0	1	4.737 ^b^		0.0014 ^c^	
2 H^+^ + L^2−^ = H_2_L^0^_(aq)_	2	6	2	0	1	7.036 ^b^		0.004 ^c^	
Na^+^ + L^2−^ = NaL^−^	1	4	0	1	1	0.99	0.08	0.0034	0.007
K^+^ + L^2−^ = KL^−^	1	4	0	1	1	0.94	0.08	−0.002	0.007
(CH_3_)_4_N^+^ + L^2−^ = (CH_3_)_4_NL^−^	1	4	0	1	1	0.50	0.12	0.01	0.01
Na^+^ + H^+^ +L^2−^ = NaHL^0^_(aq)_	2	6	1	1	1	4.75	0.11	0.001	0.01
K^+^ + H^+^ +L^2−^ = KHL^0^_(aq)_	2	6	1	1	1	4.80	0.12	−0.01	0.01
(CH_3_)_4_N^+^ + H^+^ +L^2−^ = (CH_3_)_4_NHL^0^_(aq)_	2	6	1	1	1	4.41	0.2	0.024	0.017
c_0_						0.11 ^e^			
c_1_						0.22	0.02	0.0001 ^d^	0.0012
d_1_						−0.1 ^e^			

^a^ 95%; ^b^ kept fixed during the analysis using the data from Table 3 (log *^T^*$K_{i01}^{H}$); ^c^ kept fixed during the analysis using the data obtained by dividing the ${\Delta^{T}H}_{i01}^{0\;c}$ value (Table S5) by 1702 (R·T^2^·ln(10)/1000); ^d^ A2*_ijk_*; ^e^ kept fixed during the analysis using literature values [30].

**Table 6:** Comparison of protonation constants and protonation enthalpy changes of malonic (mal), tatronic (TA), and mesoxalic (mesox) acids from literature data.

	mal ^a^	TA	mesox ^e^
log *^T^*$K_{101}^{H}$	5.70	4.74 ^b^; 4.24 ^c^; 5.37 ^d^;	3.52
log *^T^*$K_{201}^{H}$	2.85	2.30 ^b^; 2.02 ^c^; 3.69 ^d^;	1.82
ΔH1010T	4.93	−1.11 ^d^	-
ΔH2010T	−0.16	−0.49 ^d^	-

^a^ values at infinite dilution at *T* = 298.15 K [24]; ^b^ this work at infinite dilution (±0.01 at 95% C.I.); ^c^ at *T* = 293 K e *I* = 0.1 mol dm^−3^ in Na^+^ medium [24]; ^d^ at *T* = 298.15 K and *I* = 0.1 mol dm^−3^ NaClO_4_ from ref. [21]; ^e^ at *T* = 298.15 K e *I* = 0.1 mol dm^−3^ [34].

**Table 7:** Protonation constants and enthalpies of protonation for malonate and tartronate at different temperatures and ionic strengths in NaCl taken from the literature.

		mal				TA			
*T*/*K*	*I*/mol dm^−3^	$\log\;K_{101}^{H}$	$\Delta H_{101}^{0}$	$\log\;K_{102}^{H}$	$\Delta H_{102}^{0}$	$\log\;K_{101}^{H}$	$\Delta H_{101}^{0}$	$\log\;K_{102}^{H}$	$\Delta H_{102}^{0}$
298.15	0	5.67	4.93	2.84	−0.16	4.737	2.46	2.23	4.28
298.15	0.1	5.27	3.8	2.65	−0.29	4.30	1.49	2.09	3.79
298.15	0.5	5.06	2.3	2.54	−1.7	4.04	1.97	2.00	4.03
298.15	1.0	4.98	2.0	2.55	−1.5	3.93	3.19	1.98	4.64
310.15	0.15	5.24	-	2.61	-	4.24	1.18	2.08	3.64

**Table 8:** Summary of the potentiometric titrations performed on tartronic acid solutions in different ionic media at different temperatures, ionic strength values, and at *p* = 0.1 MPa.

Ionic Medium	*c*_L_ ^a^	*c_H_* ^b^	*I*/mol dm^−3^	*T*/K	n. Tit ^c^
NaCl	4.00–6.16	10.0–15.0	0.1098–0.9614	288.15, 298.15, 310.15	34
KCl	4.02–6.16	10.0–15.0	0.0977–0.1000	288.15, 298.15, 310.15	36
(CH_3_)_4_NCl	1.56–3.65	10.0–15.0	0.1657–0.9943	288.15, 298.15, 310.15	39
(C_2_H_5_)_4_NI	2.03–6.08	10.0–15.0	0.0999–0.8000	288.15, 298.15, 310.15	37

^a^ c_L_ = analytical concentration of tartronic acid (mmol dm^−3^); ^b^ analytical concentration of proton (mmol dm^−3^); ^c^ number of titration performed. Standard uncertainties (*u*): *u*(*T*) = 0.1 K; *u*(*I*) = 0.001 mol dm^−3^; *u*(p) = 1 kPa; *u*(*c*_H_) = *u*(*c*_L_) = 10^−2^ mmol dm^−3^.

## Data Availability

The original contributions presented in this study are included in the article/Supplementary Materials. Further inquiries can be directed to the corresponding author(s).

## Supplementary Materials

The following supporting information can be downloaded at: https://www.mdpi.com/article/10.3390/molecules30071497/s1, Table S1: Tartronate protonation constants values in NaCl_aq_ at different ionic strengths and temperatures in molar and molal concentration scales. Table S2: Tartronate protonation constants values in KCl_aq_ at different ionic strengths and temperatures in molar and molal concentration scales. Table S3: Tartronate protonation constants values in (CH_3_)_4_NClaq at different ionic strengths and temperatures in molar and molal concentration scales. Table S4: Tartronate protonation constants values in (C_2_H_5_)_4_NI_aq_ at different ionic strengths and temperatures in molar and molal concentration scales. Table S5: Parameters obtained fitting stepwise protonation constants to Equation (3) in the molar concentration scale (mol dm^−3^) at *T* = 298.15 K and *p* = 0.1 MPa. Table S6: Chemicals used in this work, purchased from Merck (Darmstadt, Germany). Purity (mass) is stated by the supplier. Table S7: Standard potential (in mV) and ionic product of water determined at different ionic strengths (in NaCl_aq_) and temperatures. Table S8: Standard potential (in mV) and ionic product of water determined at different ionic strengths (in KCl_aq_) and temperatures. Table S9: Standard potential (in mV) and ionic product of water determined at different ionic strengths (in (CH_3_)_4_NCl_aq_) and temperatures. Table S10: Standard potential (in mV) and ionic product of water determined at different ionic strengths (C_2_H_5_)_4_NI_aq_ and temperatures. Figure S1: (a) Dependence of ionic water product at 288.15 K in the different ionic media: ∆ (CH_3_)_4_NCl, ▽ (C_2_H_5_)_4_NI, ○ KCl, □ NaCl. (b) Dependence of ionic water product in NaCl_(aq)_ at various temperatures: □ 288.15 K, ○ 298.15 K, and ∆ 310.15 K. Refs. [28,30,32,38,39,40,41,42,43,44,45] are also cited in Supplementary Materials.
