# Supplementary material for: A Comprehensive Equilibrium Analysis of Tartronate with Proton and Major Cations in Natural Fluids
Source: Molecules. 2025 Mar 27;30(7):1497. doi: 10.3390/molecules30071497 (PMC11990633; doi:10.3390/molecules30071497)
Supplement: Supplementary file 1 [file molecules-30-01497-s001.zip › molecules-3525893-supplementary.pdf]

A Comprehensive Equilibrium Analysis of Tartronate with Proton and Major Cations in Natural Fluids

Gabriele Lando, Clemente Bretti*, Paola Cardiano, Anna Irto, Demetrio Milea and Concetta De Stefano

Department of Chemical, Biological, Pharmaceutical and Environmental Sciences, University of Messina, Viale Ferdinando Stagno d'Alcontres, 31, I-98166 Messina, Italy; glando@unime.it (G.L.); pcardiano@unime.it (P.C.); airto@unime.it (A.I.); dmilea@unime.it (D.M.); cdestefano@unime.it (C.D.S.)
* Correspondence: cbretti@unime.it (C.B.)

Ionic strength dependence according to the SIT and EDH hybrid chemico-physical models

The ionic strength dependence of the equilibrium constants was studied using an extended Debye–Hückel type equation (EDH) and Specific Ion Interaction Theory (SIT) [28, 38-40] models. For a generic formation constant, the equilibrium constants, as a function of the activity coefficients (γ), may be indicated by eq. (S1).

$$\log K_{ijk} = \log\ {}^{T}K_{ijk} + i \cdot \log \gamma_{H^+} + j \cdot \log \gamma_{X^+} + k \cdot \log \gamma_{L^{2-}} - \log \gamma_{H_iX_jL_k^{(i+j-2k)}} \qquad \text{(S1)}$$

where K_{ijk} and ${}^{T}K_{ijk}$ are the conditional and thermodynamic equilibrium constants, respectively. Apart from well-known theoretical approaches, the difference between EDH and SIT ones stems from the adopted concentration scale (c, molar for the EDH equation and m, molal for the SIT model), as well as from the nature of the $f(I)$ term:

$$\log \beta_{ijk} = \log\ {}^{T}\beta_{ijk} - \mathrm{A} \cdot z^{*} \cdot \mathrm{D.H.} + C_{ijk} \cdot I_c + D_{ijk} \cdot I_c^{3/2} + E_{ijk} \cdot I_c^{2} \quad \text{EDH approach} \qquad \text{(S2)}$$

$$\log \beta_{ijk} = \log\ {}^{T}\beta_{ijk} - \mathrm{A} \cdot z^{*} \cdot \mathrm{D.H.} + \Delta\varepsilon_{ijk} \cdot I_m \quad \text{SIT approach} \qquad \text{(S3)}$$

where C_{ijk} , D_{ijk}, and E_{ijk} are empirical parameters. Generally, for $I \leq 1$ mol dm^{-3}, as for the case of this paper, D_{ijk} and E_{ijk} parameters are set to 0, and only C_{ijk}values are refined. Under these conditions, EDH and SIT approaches become nearly identical, except for the concentration scale used.

In the classical SIT model, $\Delta\varepsilon_{ijk}$ represents the combination of specific interaction coefficients for the species involved in the equilibrium and the ions of the supporting electrolyte.

For example, in $NaCl_{(aq)}$, the SIT coefficient for the HL species becomes:

$$\Delta\varepsilon_{101} = \varepsilon(H^+, Cl^-) + \varepsilon(Na^+, L^{2-}) - \varepsilon(Na^+, HL^-) \qquad \text{(S4)}$$

Even though classical SIT only considers interactions between ions of opposite charge, neutral species can be accounted by using their activity coefficients calculated from the Setschenow coefficient (k_m). For neutral species like $H_2L^0_{(aq)}$, as in the case of tartronic acid, the interaction coefficient ε is replaced by the Setschenow constant k_m. In this case, the logarithm of the activity coefficient is defined by the following equation:

$$\log\gamma = k_m \cdot I_m \qquad \text{(S5)}$$

where k_m is the Setschenow coefficient [41].

Ionic strength dependence according to the "Pure Water" chemical model

The "Pure Water" model explains variations in the conditional protonation constants obtained in different ionic media, assuming the formation of weak ion pairs between the anionic ligand species (L^{2-} and HL^- in the case of tartronic species) and the cations of the supporting electrolyte (e.g., Na^+, K^+).

Direct measurement of these complex stabilities is often challenging, as their stability constants typically do not exceed log K = 1.0. The fundamental principles of this model have been described extensively in the literature [30], along with various examples [42, 43].

In summary, the Pure Water Model uses molar concentration [44] and relies on three key assumptions:

1. **H1**: the dependence of formation constants on ionic strength can be described by a simple equation, independent of reactants and products, and based solely on the reaction type;
2. **H2**: deviations from the expected behavior are attributed to weak complex formation between the studied species and ions in the background electrolyte (e.g., the ionic medium). This approach assumes pure water as the reference state, with some ions assumed to be non-interacting;
3. **H3**: perchlorate ions are non-interacting with cationic species. Tetraethylammonium cations (and higher homologues) do not interact with O-donor ligands, and Na^+ and K^+ ions do not interact with N-donor ligands.

These assumptions are generally valid under conditions when the ionic strength (I) is below 1 mol dm^{-3} and when $(C_2H_5)_4NI$ is used as a non-interacting medium, particularly in studies involving carboxylate ligands.

For a simple monoprotic acid (HL), the lowering effect of the "conditional" protonation constant ($\log K_{i0k}^{\mathrm{H}^{\mathrm{cond}}}$) in an "interacting" medium (e.g., NaCl) compared to a non-interacting one ($\log K_{101}^{\mathrm{H}^{\mathrm{eff}}}$) can be interpreted in terms of the formation of a weak complex between the deprotonated ligand and the cation of the supporting electrolyte (e.g., NaL at the concentration c_{Na}), whose stability constant is K_{011}:

$$\log K_{i0k}^{\mathrm{H}^{\mathrm{cond}}} = \log K_{101}^{\mathrm{H}^{\mathrm{eff}}} - \log\left(1+10^{\log K_{011}} \cdot c_{\mathrm{Na}}\right) \tag{S6}$$

For polyprotic ligands in a generic ionic medium containing an interacting M^+ cation, a slightly more complex calculation is required, but the basic assumption remains that the average number of protons bound to the ligand ($\bar{p}$) is fixed under given conditions, regardless of its expression. It can be calculated using either the "conditional" overall protonation constants ($\beta_{i0k}^{\mathrm{H}^{\mathrm{cond}}}$, referred to eq. (S1)) measured in an interacting medium

$$\bar{p}^{\,\mathrm{cond}} = \frac{\sum i \cdot \beta_{i0k}^{\mathrm{H}^{\mathrm{cond}}} [\mathrm{H}^+]^i}{1+\sum \beta_{i0k}^{\mathrm{H}^{\mathrm{cond}}} [\mathrm{H}^+]^i} \tag{S7}$$

or by using the "effective" protonation constants measured in a non-interacting medium ($\beta_{\mathrm{i0k}}^{\mathrm{H}^{\mathrm{eff}}}$) and weak complex formation constants (β_{ijk}, where $\beta_{\mathrm{i0k}} = \beta_{\mathrm{i0k}}^{\mathrm{H}^{\mathrm{eff}}}$)

$$\bar{p} = \frac{\sum i \cdot \beta_{ijk} [\mathrm{M}^+]^j [\mathrm{H}^+]^i}{1+\sum \beta_{ijk} [\mathrm{M}^+]^j [\mathrm{H}^+]^i} \tag{S7a}$$

The minimization of eq. (S7b) [45] allows the calculation of unknown formation constants of weak complexes (β_{ijk}):

$$U = \sum\left(\bar{p} - \bar{p}^{\,\mathrm{cond}}\right)^2 \qquad \text{(S7b)}$$

According to this approach, the ionic strength dependence of equilibrium constants is given as in eq. (S3), where

$$C_{ijk} = c_0 \cdot p^*_{ijk} + c_1 \cdot z^*_{ijk} \qquad \text{(S8)}$$

$$D_{ijk} = d_0 \cdot p^*_{ijk} + d_1 \cdot z^*_{ijk} \qquad \text{(S8a)}$$

$$E_{ijk} = e_0 \cdot p^*_{ijk} + e_1 \cdot z^*_{ijk} \qquad \text{(S8b)}$$

$$z^*_{ijk} = \Sigma\, z^2_{\mathrm{react}} - \Sigma\, z^2_{\mathrm{prod}} \qquad \text{(S8c)}$$

$$p^*_{ijk} = \Sigma\, p_{\mathrm{react.}} - \Sigma\, p_{\mathrm{prod.}} \qquad \text{(S8d)}$$

where c_0, c_1, d_0, d_1, e_0, and e_1 are empirical parameters valid for both protonation and complex formation equilibria; z and p are the charges and the stoichiometric coefficients of the components of the species, respectively; and β_{ijk} can be the effective protonation constant or the weak complex formation constant. Several studies [32] demonstrated that in the conditions of [H3] (i.e., in non-interacting media), the dependence of the formation constants on ionic strength fulfills [H1], and parameters c_0, c_1, and d_1 become constant (thus, eq. (S2) only depends on I, p^*_{ijk}, and z^*_{ijk}).

TABLES

Table S1. Tartronate protonation constants values in $NaCl_{aq}$ at different ionic strengths and temperatures in molar and molal concentration scales.

T/K	I/mol dm^{-3}	$\log K^{\mathrm{H}}_{101}$	s	$\log \beta^{\mathrm{H}}_{201}$	s	I/mol kg^{-1} (H_2O)	$\log K^{\mathrm{H}}_{101}$	$\log \beta^{\mathrm{H}}_{201}$
288.15	0.102	4.257	0.001	6.365	0.004	0.102	4.256	6.363
288.15	0.245	4.131	0.002	6.18	0.01	0.246	4.129	6.176
288.15	0.484	4.09	0.02	6.08	0.07	0.488	4.086	6.072
288.15	0.955	3.95	0.005	5.89	0.02	0.972	3.942	5.875
298.15	0.112	4.276	0.009	6.35	0.03	0.113	4.274	6.346
298.15	0.247	4.123	0.005	6.23	0.02	0.249	4.120	6.224
298.15	0.465	4.05	0.02	6.07	0.08	0.470	4.045	6.060
298.15	0.916	3.934	0.006	5.84	0.03	0.934	3.925	5.823
310.15	0.102	4.261	0.001	6.367	0.004	0.103	4.257	6.360
310.15	0.245	4.128	0.009	6.11	0.04	0.248	4.123	6.100
310.15	0.483	3.98	0.009	5.87	0.05	0.491	3.973	5.856
310.15	0.957	3.972	0.002	6.15	0.006	0.982	3.961	6.128

Table S2. Tartronate protonation constants values in KCl_{aq} at different ionic strengths and temperatures in molar and molal concentration scales.

T/K	I/mol dm^{-3}	log K_{101}^{H}	s	log β_{201}^{H}	s	I/mol kg^{-1} (H_2O)	log K_{101}^{H}	log β_{201}^{H}
288.15	0.101	4.240	0.002	6.23	0.01	0.101	4.238	6.227
288.15	0.246	4.20	0.03	6.1	0.1	0.248	4.197	6.094
288.15	0.479	4.084	0.002	5.92	0.01	0.486	4.078	5.908
288.15	0.964	4.048	0.006	6.1	0.02	0.991	4.036	6.076
298.15	0.094	4.29	0.01	6.43	0.06	0.095	4.288	6.425
298.15	0.246	4.150	0.001	6.228	0.003	0.248	4.146	6.220
298.15	0.484	4.03	0.01	6.06	0.05	0.492	4.023	6.046
298.15	0.961	3.97	0.03	5.8	0.2	0.990	3.957	5.774
310.15	0.102	4.30	0.01	6.48	0.05	0.103	4.296	6.472
310.15	0.246	4.153	0.002	6.243	0.006	0.249	4.147	6.231
310.15	0.483	4.055	0.001	6.069	0.006	0.493	4.046	6.051
310.15	0.959	4.015	0.002	6.103	0.008	0.993	4.090	6.163

Table S3. Tartronate protonation constants values in $(CH_3)_4NCl_{aq}$ at different ionic strengths and temperatures in molar and molal concentration scales.

T/K	I/mol dm^{-3}	log K_{101}^{H}	s	log β_{201}^{H}	s	I/mol kg^{-1} (H_2O)	log K_{101}^{H}	log β_{201}^{H}
288.15	0.165	4.25	0.01	6.28	0.03	0.168	4.242	6.264
288.15	0.323	4.247	0.009	6.32	0.02	0.335	4.231	6.289
288.15	0.531	4.227	0.004	6.36	0.02	0.564	4.201	6.308
288.15	0.954	4.233	0.005	6.36	0.02	1.064	4.186	6.266
298.15	0.168	4.243	0.001	5.93	0.004	0.172	4.234	5.913
298.15	0.323	4.23	0.001	6.28	0.007	0.336	4.226	6.207
298.15	0.539	4.17	0.01	5.88	0.04	0.574	4.143	5.825
298.15	0.728	4.254	0.006	6.34	0.02	0.792	4.217	6.266
298.15	0.958	4.23	0.02	6.61	0.09	1.071	4.181	6.513
310.15	0.165	4.287	0.001	6.371	0.005	0.169	4.276	6.349
310.15	0.319	4.212	0.001	6.22	0.003	0.333	4.194	6.183
310.15	0.48	4.231	0.005	6.31	0.01	0.510	4.205	6.258
310.15	0.957	4.241	0.004	6.32	0.02	1.075	4.190	6.219

Table S4. Tartronate protonation constants values in $(C_2H_5)_4NI_{aq}$ at different ionic strengths and temperatures in molar and molal concentration scales.

T/K	I/mol dm^{-3}	$\log K_{101}^{H}$	s	$\log \beta_{201}^{H}$	s	I/mol kg^{-1} (H_2O)	$\log K_{101}^{H}$	$\log \beta_{201}^{H}$
288.15	0.103	4.46	0.03	6.58	0.07	0.105	4.451	6.563
288.15	0.246	4.378	0.001	6.535	0.004	0.258	4.358	6.494
288.15	0.484	4.342	0.005	6.55	0.02	0.532	4.301	6.469
288.15	0.618	4.393	0.003	6.51	0.01	0.697	4.340	6.405
288.15	0.767	4.41	0.01	6.58	0.03	0.893	4.344	6.448
298.15	0.102	4.394	0.002	6.569	0.006	0.104	4.384	6.550
298.15	0.246	4.353	0.001	6.494	0.002	0.258	4.332	6.451
298.15	0.483	4.427	0.001	6.684	0.001	0.532	4.385	6.600
298.15	0.764	4.451	0.002	6.628	0.006	0.892	4.384	6.494
298.15	0.572	4.434	0.001	6.653	0.004	0.641	4.384	6.554
310.15	0.102	4.44	0.001	6.718	0.006	0.105	4.429	6.696
310.15	0.242	4.421	0.001	6.684	0.005	0.255	4.398	6.638
310.15	0.483	4.339	0.001	6.526	0.005	0.534	4.295	6.438
310.15	0.764	4.474	0.003	6.67	0.008	0.896	4.405	6.531

Table S5. Parameters obtained fitting stepwise protonation constants to eq. (3) in the molar concentration scale (mol dm^{-3}) at T = 298.15 K and p = 0.1 MPa.

			**Molar concentration scale (EDH)**				
			NaCl	KCl	$(CH_3)_4NCl$	$(C_2H_5)_4NI$	
i	$\log {}^{T}K_i^{H}$	${}^{T}\Delta H_{i01}^{0}$	C_{i01}				C'_{MX}
1	4.74 ± 0.01 [a]	2.5 ± 1.8 [a]	0.01 ± 0.02 [a]	0.09 ± 0.03 [a]	0.33 ± 0.02 [a]	0.67 ± 0.02 [a]	3.1 ± 3.1 [a]
2	2.30 ± 0.01	4.3 ± 1.3	0.09 ± 0.04	0.10 ± 0.03	0.23 ± 0.02	0.44 ± 0.03	1.6 ± 1.6

[a] ± s (standard deviation); standard uncertainties (u): $u(T)$ = 0.1 K; $u(p)$ = 1 kPa.

Table S6. Chemicals used in this work, purchased from Merck (Darmstadt, Germany). Purity (mass) is stated by the supplier.

**Chemical**	**Formula**	**CAS n°**	**Purification**	**Assay (mass)**
Sodium chloride	NaCl	7647-14-5	NO	≥ 99%
Potassium chloride	KCl	7447-40-7	NO	≥ 99%
Tetraethylammonium iodide	$(C_2H_5)_4NI$	68-05-3	recrystallisation	98%
Tetramethylammonium chloride	$(CH_3)_4NCl$	75-57-0	recrystallisation	≥98%
Hydrochloric acid	HCl	7647-01-0	NO	≥ 99%
Tetraethylammonium hydroxide	$(C_2H_5)_4NOH$	77-98-5	NO	~10% [a]
Tetramethylammonium hydroxide	$(CH_3)_4NOH$	10424-65-4	NO	~10% [a]
Potassium hydroxide concentrate (0.1 mol dm^{-3})	KOH	1310-58-3	NO	
Sodium hydroxide	NaOH	1310-73-2	NO	≥ 99%
Potassium phthalate monobasic	$C_8H_5O_4K$	877-24-7	NO	≥ 99.95%
Sodium carbonate	Na_2CO_3	497-19-8	NO	99.995 %
Tartronic acid	$C_3H_4O_5$	80-69-3	NO	≥ 97%

[a] Value refers to the concentration in the solutions. On the dry basis, the assay is ≥ 99.5 % (on the mass basis).

Table S7. Standard potential (in mV) and ionic product of water determined at different ionic strengths (in $NaCl_{aq}$) and temperatures.

T/K	I/mol dm^{-3}	E^0	s	log K_w	s
288.15	0.102	366.47	0.03	-14.0841	0.0006
288.15	0.103	366.58	0.02	-14.0908	0.0004
288.15	0.104	366.16	0.02	-14.0855	0.0008
288.15	0.241	365.58	0.04	-14.0482	0.0008
288.15	0.242	365.83	0.03	-14.0455	0.0008
288.15	0.244	365.74	0.02	-14.0432	0.0008
288.15	0.473	367.72	0.03	-14.0211	0.0005
288.15	0.474	365.78	0.04	-14.0128	0.0008
288.15	0.475	367.4	0.03	-14.019	0.001
288.15	0.933	373.11	0.09	-14.0328	0.0002
288.15	0.934	373.72	0.02	-14.047	0.001
288.15	0.935	373.76	0.06	-14.04	0.02
298.15	0.112	411.4	0.1	-13.631	0.003
298.15	0.107	412.2	0.1	-13.694	0.003
298.15	0.248	407.86	0.04	-13.699	0.005
298.15	0.465	400.17	0.04	-13.654	0.004
298.15	0.464	400.79	0.02	-13.628	0.005
298.15	0.46	401.07	0.03	-13.661	0.003
298.15	0.925	404.37	0.05	-13.614	0.007
298.15	0.914	404.15	0.07	-13.651	0.006
298.15	0.904	404.5	0.03	-13.715	0.002
310.15	0.102	394.97	0.04	-13.3594	0.0008
310.15	0.099	396.34	0.09	-13.410	0.01
310.15	0.101	393.88	0.03	-13.387	0.001
310.15	0.241	396.98	0.02	-13.3407	0.0008
310.15	0.221	397.49	0.04	-13.3693	0.0008
310.15	0.243	395.06	0.06	-13.339	0.001
310.15	0.473	398.8	0.1	-13.310	0.002
310.15	0.474	394.95	0.08	-13.247	0.002
310.15	0.475	394.79	0.06	-13.256	0.002
310.15	0.936	405.22	0.05	-13.329	0.002
310.15	0.936	403.9	0.1	-13.323	0.002
310.15	0.941	402.82	0.05	-13.335	0.001

Table S8. Standard potential (in mV) and ionic product of water determined at different ionic strengths (in KCl_{aq}) and temperatures.

T/K	I/mol dm^{-3}	E^0	s	log K_w	s
288.15	0.102	370.0	0.2	-14.138	0.003
288.15	0.103	366.3	0.1	-14.094	0.002
288.15	0.104	368.28	0.09	-14.123	0.002
288.15	0.241	366.42	0.05	-14.0667	0.0009
288.15	0.242	366.94	0.02	-14.0817	0.0005
288.15	0.243	366.38	0.09	-14.071	0.002
288.15	0.473	367.49	0.03	-14.0729	0.0008
288.15	0.474	367.23	0.03	-14.0700	0.0006
288.15	0.475	366.88	0.02	-14.0669	0.0004
288.15	0.937	369.108	0.002	-14.1127	0.0005
288.15	0.938	367.93	0.05	-14.0976	0.0001
288.15	0.940	367.8	0.1	-14.098	0.002
298.15	0.102	385.39	0.01	-13.7966	0.0005
298.15	0.094	382.80	0.02	-13.7999	0.0005
298.15	0.241	381.92	0.02	-13.7583	0.0003
298.15	0.242	382.08	0.01	-13.7610	0.0002
298.15	0.244	381.824	0.005	-13.7605	0.0003
298.15	0.473	381.78	0.03	-13.738	0.001
298.15	0.474	381.64	0.02	-13.7470	0.0004
298.15	0.937	383.76	0.03	-13.7797	0.0006
298.15	0.938	383.51	0.02	-13.7715	0.0004
298.15	0.939	383.62	0.01	-13.7743	0.0006
310.15	0.102	395.48	0.05	-13.4003	0.0008
310.15	0.103	396.50	0.02	-13.4155	0.0004
310.15	0.104	395.33	0.03	-13.4221	0.0005
310.15	0.241	393.87	0.02	-13.3700	0.0004
310.15	0.242	393.63	0.02	-13.3781	0.0005
310.15	0.244	392.09	0.02	-13.3752	0.0004
310.15	0.473	392.11	0.02	-13.3533	0.0004
310.15	0.475	378.16	0.09	-13.395	0.002
310.15	0.476	379.33	0.05	-13.404	0.001
310.15	0.938	398.41	0.06	-13.391	0.001
310.15	0.939	399.06	0.02	-13.4103	0.0004
310.15	0.940	398.10	0.04	-13.405	0.001

Table S9. Standard potential (in mV) and ionic product of water determined at different ionic strengths (in $(CH_3)_4NCl_{aq}$) and temperatures.

T/K	*I*/mol dm^{-3}	E^0	s	log K_w	s
288.15	0.325	363.84	0.09	-14.078	0.007
288.15	0.166	364.13	0.08	-14.084	0.005
288.15	0.166	364.13	0.03	-14.081	0.003
288.15	0.959	364.4	0.1	-14.12	0.01
288.15	0.533	364.16	0.09	-14.059	0.009
298.15	0.166	374.15	0.06	-	-
298.15	0.325	375.58	0.03	-13.45	0.02
298.15	0.535	375.37	0.06	-13.53	0.02
298.15	0.960	376.97	0.08	-13.41	0.04
298.15	0.727	377.13	0.04	-13.39	0.03
310.15	0.948	392.76	0.06	-13.507	0.003
310.15	0.166	392.83	0.04	-13.4351	0.0008
310.15	0.478	392.21	0.05	-13.448	0.002
310.15	0.478	392.37	0.06	-13.442	0.002
310.15	0.323	392.3	0.07	-13.432	0.002

Table S10. Standard potential (in mV) and ionic product of water determined at different ionic strengths $(C_2H_5)_4NI_{aq}$ and temperatures.

T/K	*I*/mol dm^{-3}	E^0	s	log K_w	s
288.15	0.102	366.17	0.03	-14.1633	0.0006
288.15	0.103	366.10	0.02	-14.1588	0.0007
288.15	0.241	366.72	0.07	-14.183	0.001
288.15	0.242	366.71	0.03	-14.178	0.001
288.15	0.244	366.45	0.05	-14.158	0.003
288.15	0.473	366.93	0.07	-14.245	0.001
288.15	0.474	366.72	0.06	-14.234	0.001
288.15	0.475	366.51	0.03	-14.216	0.001
288.15	0.753	367.53	0.06	-14.294	0.002
288.15	0.753	367.30	0.04	-14.321	0.001
288.15	0.604	366.90	0.05	-14.029	0.001
298.15	0.102	379.58	0.04	-13.8330	0.0008
298.15	0.103	379.62	0.03	-13.8337	0.0008
298.15	0.104	379.54	0.02	-13.830	0.001
298.15	0.241	379.31	0.03	-13.8535	0.0005
298.15	0.242	379.18	0.03	-13.8305	0.0009
298.15	0.243	382.51	0.02	-13.9672	0.0007
298.15	0.472	382.65	0.02	-14.0332	0.0004
298.15	0.475	382.92	0.04	-14.0109	0.0007
298.15	0.753	383.03	0.02	-14.1122	0.0009
298.15	0.569	383.072	0.001	-14.0259	0.0002
310.15	0.102	397.55	0.08	-13.563	0.002
310.15	0.103	397.74	0.04	-13.5653	0.0007
310.15	0.104	398.194	0.009	-13.5719	0.0007
310.15	0.241	398.41	0.02	-13.5978	0.0006
310.15	0.243	388.54	0.02	-13.604	0.001
310.15	0.472	388.92	0.05	-13.664	0.001
310.15	0.473	398.13	0.04	-12.6605	0.0009
310.15	0.75	398.65	0.08	-13.753	0.001
310.15	0.753	398.89	0.06	-13.732	0.006

FIGURE

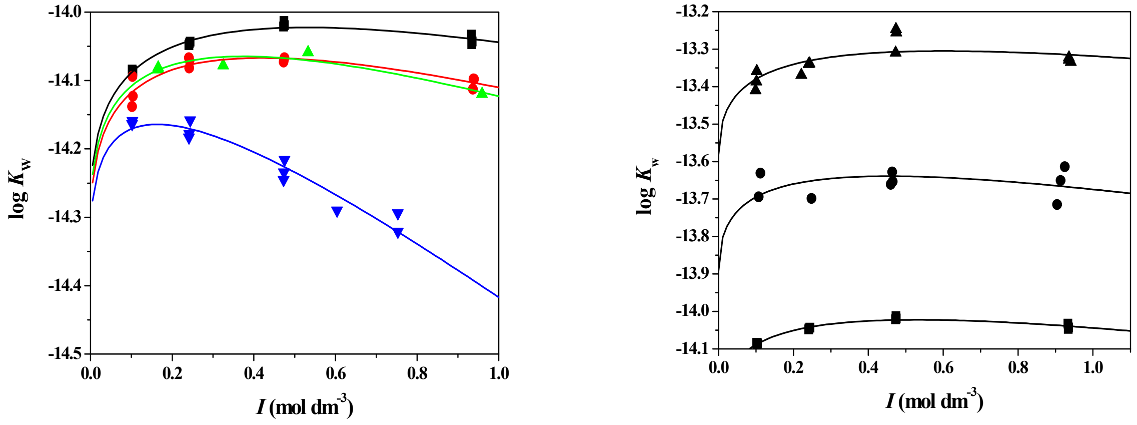

(a) (b)

Figure S1. (a) Dependence of ionic water product at 288.15 K in the different ionic media: Δ $(CH_3)_4NCl$, ∇ $(C_2H_5)_4NI$, ○ KCl, □ NaCl. (b) Dependence of ionic water product in $NaCl_{(aq)}$ at various temperatures: □ 288.15 K, ○ 298.15 K, and Δ 310.15 K.

In the analysis of Figure S1a, it can be observed that the ionic product of water increases up to a certain ionic strength in different media but then decreases as the ionic strength continues to rise. This variation is more pronounced in the calibration measurements carried out in NaCl. On the other hand, Figure S1b demonstrates that, within the same ionic medium, the ionic product of water increases with rising temperatures.

References (see Manuscript list)

28. Bretti, C.; Foti, C.; Porcino, N.; Sammartano, S., SIT Parameters for 1:1 Electrolytes and Correlation with Pitzer Coefficients. *J. Sol. Chem.* **2006,** 35, (10), 1401-1415.
30. Daniele, P. G.; Foti, C.; Gianguzza, A.; Prenesti, E.; Sammartano, S., Weak alkali and alkaline earth metal complexes of low molecular weight ligands in aqueous solution. *Coord. Chem. Rev.* **2008,** 252, (10), 1093-1107.
32. Castellino, L.; Alladio, E.; Bertinetti, S.; Lando, G.; De Stefano, C.; Blasco, S.; García-España, E.; Gama, S.; Berto, S.; Milea, D., PyES – An open-source software for the computation of solution and precipitation equilibria. *Chemometr. Intell. Lab. Syst.* **2023,** 239, 104860.
38. Brønsted, J., Studies on solubility. IV. The principle of the specific interaction of ions. *J. Am. Chem. Soc.* **1922,** 44, (5), 877-898.
39. Ciavatta, L., The specific interaction theory in evaluating ionic equilibria. *Ann. Chim.(Rome)* **1980,** 70, 551.
40. Guggenheim, E.; Turgeon, J., Specific interaction of ions. *J. Chem. Soc. Faraday Trans.* **1955,** 51, 747-761.
41. Setschenow, J. Z., Uber Die Konstitution Der Salzlosungenauf Grund Ihres Verhaltens Zu Kohlensaure. *Z. Physik. Chem.* **1889**, (4), 117-125.
42. Cigala, R. M.; Raccuia, S. G. M.; Bretti, C.; Cardiano, P.; Lando, G.; Gomez Laserna, O. ; Gattuso, G.; Irto, A.; Crea, F.; De Stefano, C., Gallic acid as potential sequestering agent for methylmercury(II) and dimethyltin(IV) removal from aqueous solutions. *J. Environ. Chem. Eng.* **2024,** 12, (6), 114620.
43. Bretti, C.; Cigala, R. M.; De Stefano, C.; Lando, G.; Milea, D.; Sammartano, S., On the interaction of phytate with proton and monocharged inorganic cations in different ionic media, and modeling of acid-base properties at low ionic strength. *J. Chem. Thermodyn.* **2015,** 90, 51-58.
44. Pytkowicz, R. M., Activity coefficients in electrolyte solutions*; CRC Press: Boca Raton, FL, USA,* **1979**.
45. Cigala, R. M.; Cordaro, M.; Crea, F.; De Stefano, C.; Fracassetti, V.; Marchesi, M.; Milea, D.; Sammartano, S., Acid–Base Properties and Alkali and Alkaline Earth Metal Complex Formation in Aqueous Solution of Diethylenetriamine-N,N,N′,N″,N″-pentakis(methylenephosphonic acid) Obtained by an Efficient Synthetic Procedure. *Ind. Eng. Chem. Res.* **2014,** 53, (23), 9544-9553.
